# The long-term arterial assist intermittent pneumatic compression generating venous flow obstruction is responsible for improvement of arterial flow in ischemic legs

**DOI:** 10.1371/journal.pone.0225950

**Published:** 2019-12-11

**Authors:** Marzanna T. Zaleska, Waldemar L. Olszewski, Jonathan Ross

**Affiliations:** 1 Department of Applied Physiology, Mossakowski Medical Research Center, Polish Academy of Sciences, Warsaw, Poland; 2 Central Clinical Hospital, Ministry of Internal Affairs, Department of Surgery, Warsaw, Poland; 3 Lehigh University, Philadelphia, PA, United States of America; Jagiellonian University Medical College, POLAND

## Abstract

**Background:**

There is a large group of patients with ischemia of lower limbs not suitable for surgical reconstruction of arteries treated with the help of external assist by intermittent pneumatic compression devices (IPC). Until recently the generally accepted notion was that by compressing tissues below the knee, veins become emptied, venous pressure drops to zero and the increased arterial-venous pressure gradient enables greater arterial flow. We used a pump that, in contradiction to the “empty veins” devices, limited the limb venous outflow by venous obstructions and in a long period therapy expanded the perfusion vessels and brought about persistent reactive hyperemia.

**Aim:**

To check the toe and calf arterial inflow measured by venous stasis plethysmography and capillary flow velocity during arterial assist IPC in a long-term therapy of ischemic legs.

**Material and methods:**

Eighteen patients (12M, 6F) age 62 to 75 with leg peripheral arterial disease (PAD, Fontaine stage II) were studied. Pneumatic device with two 10cm wide cuffs (foot, calf) (Bio Compression Systems, Moonachie, NJ, USA) inflated to 120 mmHg for 5–6 sec to obstruct the venous flow, deflation time 16 sec, applied for 45–60 min daily for a period of 2 years.

**Results:**

At pump inflation increase in toe arterial pressure, volume, capillary blood flow velocity and one-minute arterial inflow test was observed. Increased toe volume appeared concomitantly with the inflated chamber venous obstruction. Resting pressure in the great saphenous vein increased. The two years therapy showed persistence of the resting limb increased toe capillary flow. Intermittent claudication distance increased by 20–120%. After two years arterial assist TBI increased from 0.2 to 0.6 (range 0.3 to 0.8) (p<0.05 vs pre-therapy). The toe arterial inflow dominated over that in calf skin and muscles, nevertheless, there was prolongation of the claudication distance presumably due to dilatation of exchange vessels also in muscles.

**Conclusions:**

Our arterial assist IPC brought about increase in the toe capillary flow, long lasting dilatation of toe capillaries and extension of painless walking distance. The crucial factor of rhythmic repeated venous outflow obstructions should be taken into account in designing effective assist devices.

## Introduction

Ischemia of lower limbs due to atherosclerosis of arteries causing intermittent claudication, and in cases of more advanced state necrotic changes of peripheral tissues, concerns in the age group above 60 years between 1% and 7% of the population. Pharmacological treatment, especially with statins, limited the indications for surgical treatment, created a challenge for more effective conservative treatment. There is a large group of patients with ischemia of the lower leg and foot without effect of the pharmacological treatment who are not eligible for surgical reconstruction of the arteries due multi-segmental arterial obstruction and general contraindications for major surgery. The only treatment here is increasing blood peripheral tissue flow with the help of external support by intermittent pneumatic compression devices (IPC) [1]. There is no objective data, except hypotheses from the academic centers, documenting the mechanism of increasing the arterial flow and increasing the knowledge of the beneficial effects [2].Despite of IPC technology having been available in its present form for many years uncertainty remains about its mechanism to improve arterial inflow and clinical effectiveness in a population who are in urgent need of effective treatment options [3]. Attempts to support the inflow of arterial blood to ischemic limb regions by means of pneumatic pumps and sleeves have a long tradition [4–15].

There are several mechanisms by which a positive effect on the lower limb tissue perfusion is conferred by the use of IPC. These include emptying of the plantar venous plexus, reduction of the venous leg pressure, increase of the arterio-venous pressure gradients in dependent patients, increase of arterial flow, release of vasodilators (nitric oxide, prostacyclins), reduction of local vascular resistance, induction of post occlusive hyperemia and transient suspension of the arteriolar-venous reflex [16–20].

Until recently the generally accepted notion was that by compressing all the tissues below the knee by an inflated cuff, a large volume of venous blood is emptied with venous pressure dropping to nearly zero. The increased arterial-venous pressure gradient results in a greater arterial flow. The applied device pressures were set below arterial pressure so that arterial pressures remained unaffected. The concluding remarks were that “IPC foot-calf is the most effective in emptying the leg veins” [9]. It was reported that using rapid IPC with pressures of around 100 mmHg tripled popliteal artery flow, demonstrating significant decrease in peripheral arterial resistance caused by reduction of venous pressure. It may be presumed that in these studies rapid 0.6 sec inflation of chambers a strike transfer of force generated venous-arteriolar reflex (VAR) with subsequent stop to arterial inflow and vein filling.

The quoted interpretation of the IPC mechanism should be challenged by observation of patients with night pains alleviated by changing supine to sitting leg position increasing venous pressure and volume but not so much arterial limited by atherosclerotic obstruction [21]. It was found that patients with relief of pain while sitting did have a higher capillary density in the sitting position [22, 23]. Our concept was to follow this observation and apply a slow inflating pump that, due to the IPC long-term repeated venous stasis episodes PC may lead to a persistent dilatation of tissue capillaries. A slow inflation would diminish the venous-arterioral reaction. The retrograde hydrostatic dilatation of microcirculation at the venous end of capillaries will be followed by increase of the capillary exchange surface, slower perfusion, more time for oxygen extraction and absorption of waste products. In this way the arterial flow assist pump limiting venous outflow would mimick the events in veins during changing leg position from the supine to the upright.

In this study we investigated the mechanism of the designed arterial assist pump generating intermittent venous flow obstruction on the toe and calf arterial pulse, toe capillary flow velocity and arterial inflow volume as well as the intermittent claudication distance at two time points, after 1 hour and 2 years daily arterial flow assists.

## Material and methods

### Clinical setting

Eighteen patients (12M, 6F) age 62 to 75 with leg peripheral arterial disease (PAD) were studied. They were admitted in an order as they showed up for the out-patients clinic check-up. The follow-up period was 5 years starting 2013 until 2018. Inclusion criteria: patients suffering from leg claudication below 100m (Fontaine II), multifocal partial or total obstruction of femoral or calf arteries on arteriography ineligible for surgical revascularization or percutaneous transluminal angioplasty (PTA) due to systemic contraindications. Exclusion criteria: foot inflammation and necrosis, superficial or deep venous thrombosis, the post-thrombotic syndrome, limb post-trauma edema, rheumatic arthritis, previous arterial reconstruction and cardiac arrhythmia. No anticoagulants or vasodilating drugs were taken. Verbal inform consent was given by the patients. The study received consent of the Warsaw Medical University ethics committee in 2013. It waived the need for written consent as each admitted patient signs hospital form with agreement on undergoing the necessary test and therapy.

### Pneumatic arterial assist pump

Pneumatic device with two 10cm wide cuffs (foot, calf) (Bio Compression Systems, Moonachie, NJ, USA) slowly inflated to 120 mmHg for 5–6 sec to occlude the venous flow, deflation time 16 sec. Delay between foot and calf inflation was 1 sec. Pump was applied daily for 45–60 min for a period of over 2 years. At the initial stage, a two-weeks teaching session was set in our out-patients clinic with instruction on pump use and claudication recording. Intermittent controls were carried out every 3 months. Compliance rates measured by both adherence (to the daily treatment sessions) and persistence (duration of compliance with the course of treatment) were 95%.

### Big-toe arterial pressure measurement

Briefly, a plethysmograph (Hokanson, Bellevue, WA, type EC6) in a recording arterial mode was applied. The sensor was attached to the posterior aspect of the big toe.

### Continuous point Doppler capillary flow velocity in the big toe and calf skin

**Fig 1:**
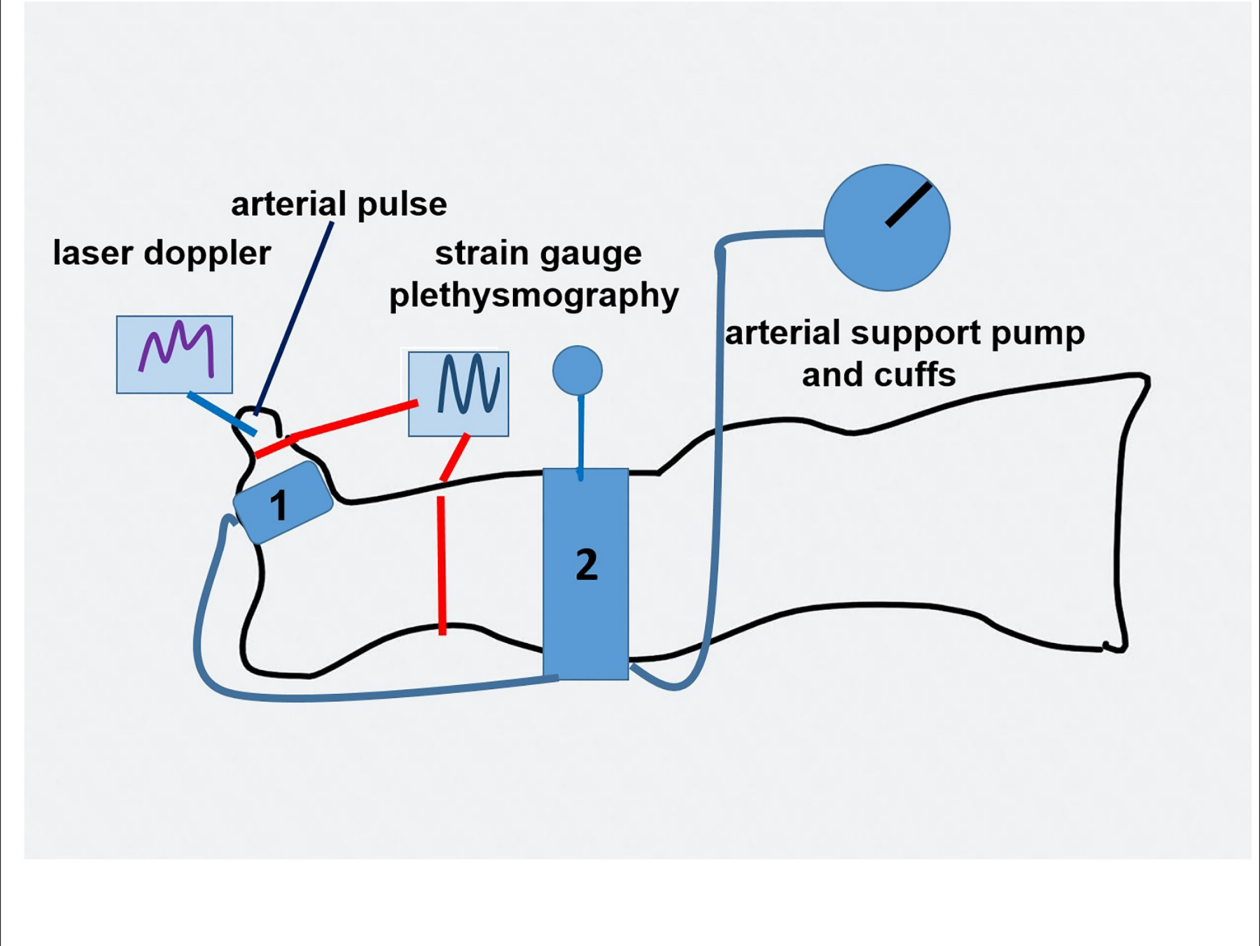
Sites for recording the toe skin capillary flow and arterial pulse, and arterial inflow into toe and calf. Numbers 1 and 2 depict arterial assist pump chambers.

This method was applied to follow changes in the toe and calf skin capillary amplitude (red cell density and movement velocity): a) during arterial assist and b) for measuring arterial closing pressure by gradual venous obstruction at 50, 80, 100 and 120 mmHg before and after each arterial assist cycle. Disappearance of capillary pulse wave was considered as arterial closing pressure. A single-channel laser Doppler flow meter was used with a specialized fiber optic probe to measure blood cell perfusion in the microvasculature of tissues (Relative Red Blood Cell flux) (Fig 1). Class 1 Laser (as per 21 CFR 1040–10 and 1040–11). To obtain the resting capillary flow velocity data the investigated subjects remained in a horizontal position with uncovered limbs, motionless, at room temperature for 15 minutes.

### Continuous big toe and calf circumference and volume measurements

The rate of blood inflow to the big toe and calf was measured by plethysmography. Briefly, a plethysmograph (Hokanson, Bellevue, WA, type EC6) in a recording vein mode was applied (Fig 1). Mercury strain gauges of a length of 7.5 cm and 32 cm were put around the big toe and mid-calf. Elongation of the gauge was read off on the recorder graph scale in mm. It showed increase in circumference brought about by the in-flowing blood. For measuring of arterial inflow (venous volume) a fast 5 seconds (filling of large veins) and slow 55 seconds (distension of venous capillaries and plasma filtration) arterial inflow phase curve was recorded. Sequential venous obstruction pressures from 50 to 150mmHg were used to follow the maximum toe and calf venous (arterial inflow) volume changes. The truncated cone formula for measuring toe and calf volumes changes was applied before and after arterial assist.

### Great saphenous vein pressure recording

Under local anesthesia the 18-gauge needle connected to the pressure transducer (Honeywell, Elblinger, Poland) was introduced to the vein. Recording was done on a device with with pressure range -20 to +150 mmHg (Telsoft, Warsaw, Poland) and using LabView software (National Instruments, Austin, TX, USA). Position of the transducer was zeroed placing it exactly at the level of the subcutaneously located needle. The data were collected using Microsoft Excel program and were presented graphically on a pressure/time scale.

### Statistical evaluation

Comparison between the pre- and post-therapy data was done using the double-tail Student test with significance at P<0.05.

## Results

### The blood flow measurement before and after arterial assist one hour session

#### Big-toe arterial pulse

**Fig 2:**
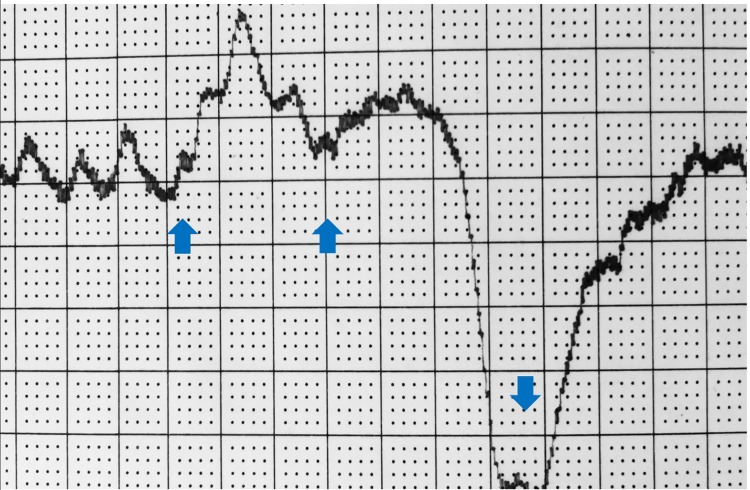
The big toe arterial pulse recording during arterial pump support. First arrow at inflation time of chamber 1 (fore-foot), second of chamber 2 (mid-calf) and third deflation of both chambers. Note increase in pulse amplitude immediately after chambers inflation due to a 4–6 sec toe arterial dilatation, before chamber pressure reaches 120mmHg causing a 2 sec drop due to stop of arterial inflow.

The pulse amplitude increased immediately after IPC chambers inflation for 5–6 sec by 10-15mmHg, before the inflating pressure reached 120mmHg bringing about a 2 sec drop of arterial inflow and pressure (Fig 2). This meant that obstruction of the venous outflow produced a few seconds lasting blood accumulation in the veins and in a retrograde fashion the supplying arteries.

### Toe and calf skin capillary Doppler recordings

#### Toe Doppler capillary red cells flow velocity during single pump inflation

**Fig 3:**
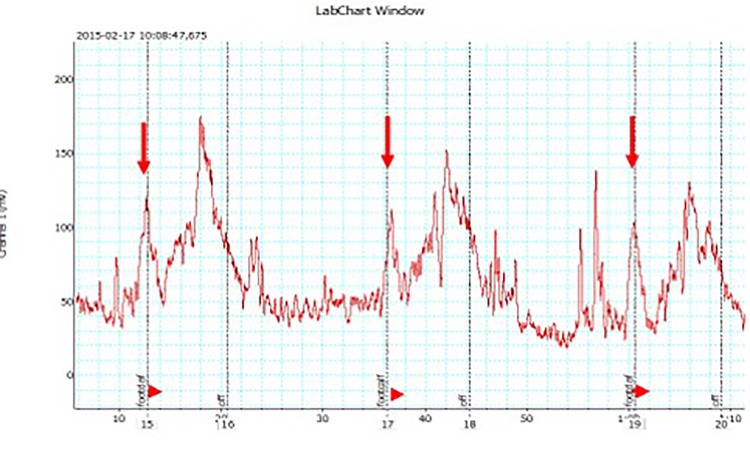
Laser Doppler recording of the big toe capillary flow velocity (in millivolts) during the designed arterial assist pump (arrows point 4–6 sec inflation of foot-calf chambers) in a patient with multifocal calf arteries occlusions. The peak is followed by a short drop and then increase in flow returning to control values during the 16 sec (off) without compression.

Arterial assist pump inflation was followed by immediate increase in the toe capillary flow velocity in a range from 10 mV to 300 mV depending on the patient, then a slight drop for 2 sec followed by increase to values higher than before pump inflation (Fig 3). Similar recordings were obtained in all patients. Interestingly, in contrast to the toe, calf skin capillary flow did not show evident pre- and post-IPC differences.

#### Toe capillary Doppler red cell velocity after 10 minutes arterial assist inflations

**Fig 4:**
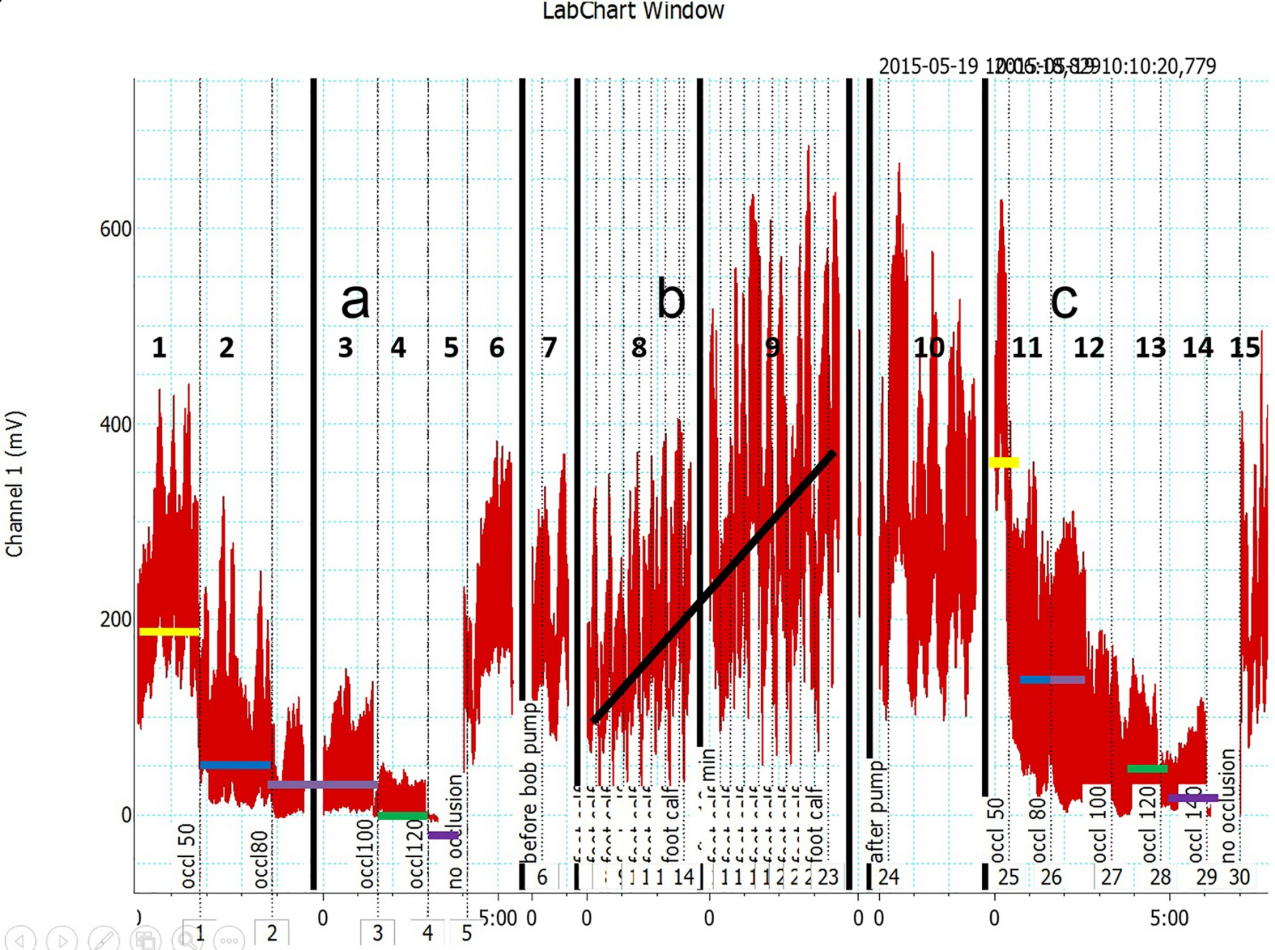
Laser Doppler recording of the big toe capillary flow velocity (in millivolts) part a) before (points 1–5), no assist (points 6–7), part b) during (points 8–9), no assist (point 10), part c) after (points 11–14), and no assist (point 15) Biocompression arterial pump application in a patient with multifocal calf arteries occlusions. In order to observe the pump effect on the capillary flow before and after IPC the venous occlusion plethysmographic method was used at venous occlusion pressures from 50 through 80, 100, 120 and 140 mmHg, before (points 1–5) and after (points 11–14) 20 arterial pump inflations. Horizontal lines at these points denote mean values. Oblique line in part b point 8 and 9 shows values during pump inflations. Note that capillary flow velocity remained increased after pump assist. Comparison of the pre- and post-IPC capillary flow velocity values showed their higher values after assist even at the plethysmographic arterial inflow occlusion as high as 140 mmHg.

**Fig 5:**
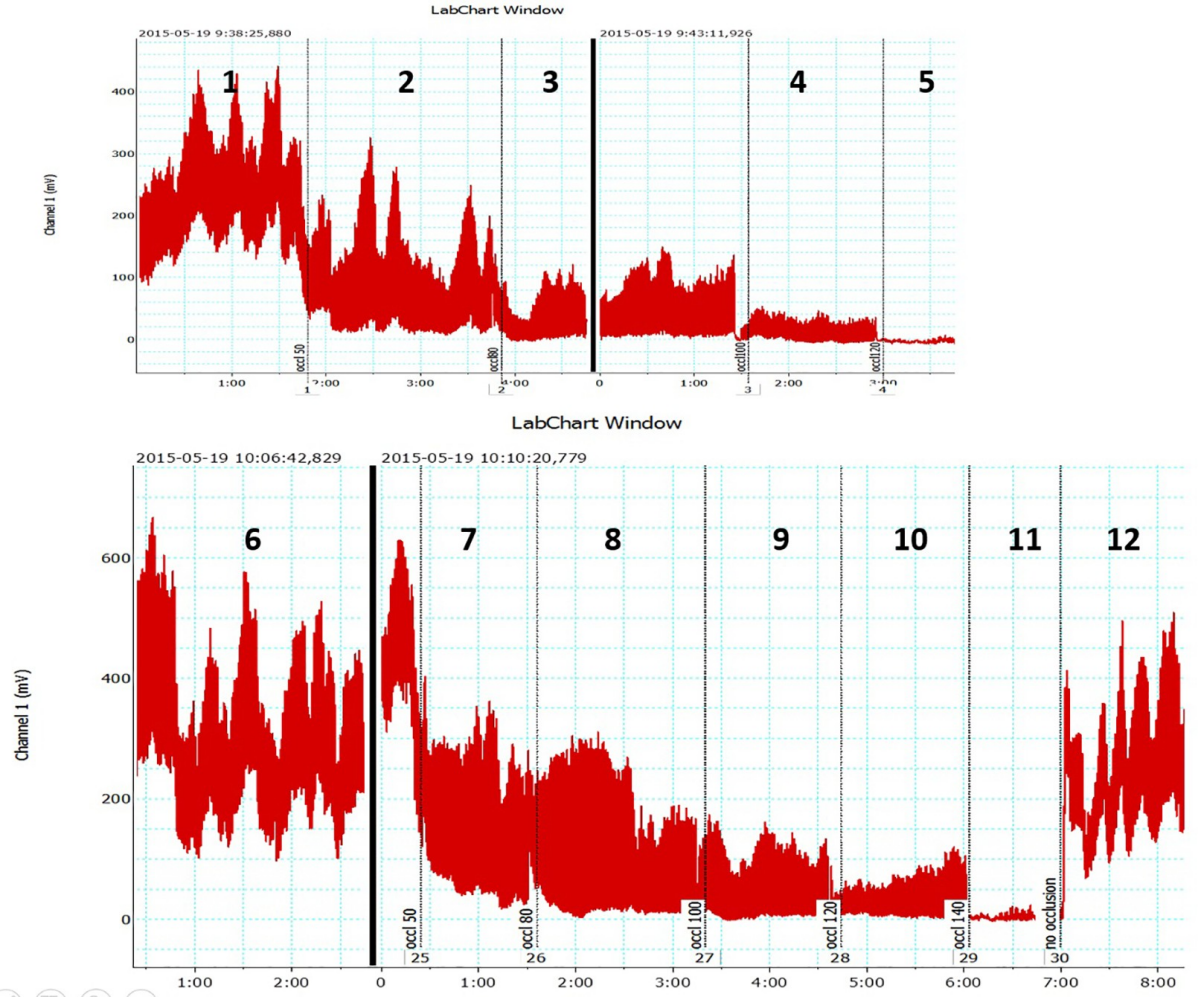
Laser Doppler recording of the big toe capillary flow velocity (in millivolts) before (upper part) and after (lower part) Biocompression arterial pump assist in a patient with multifocal calf arteries occlusions. Control value (point 1). In order to observe the pump effect on the capillary flow before and after pump assist the venous occlusion plethysmographic method was used at venous occlusion pressures from 50 through 80, 100, 120 and 140 mmHg, before (points 2–5) and after (points 7–11) arterial pump inflations. Comparison of the pre- and post-IPC capillary flow velocity values showed higher values after support even at the plethysmographic arterial inflow occlusion of 140 mmHg.

The pre-assist venous obstruction plethysmography of 50 to100 mmHg showed decreased capillary flow with occlusion at 120mmHg in most investigated subjects (Fig 4). The assist pump inflation pressure increased the capillary flow velocity. The post-assist plethysmography showed the capillary velocity still present at the occlusion pressure of 120mmHg, recording not seen before he assist. The difference between the pre- and post-IPC was observed in all patients (p<0.05). In some patients plethysmography showed capillary flow after arterial assist even at the occlusion pressure as high as 140mmHg (Fig 5).

### The toe and calf circumference during arterial support

**Fig 6:**
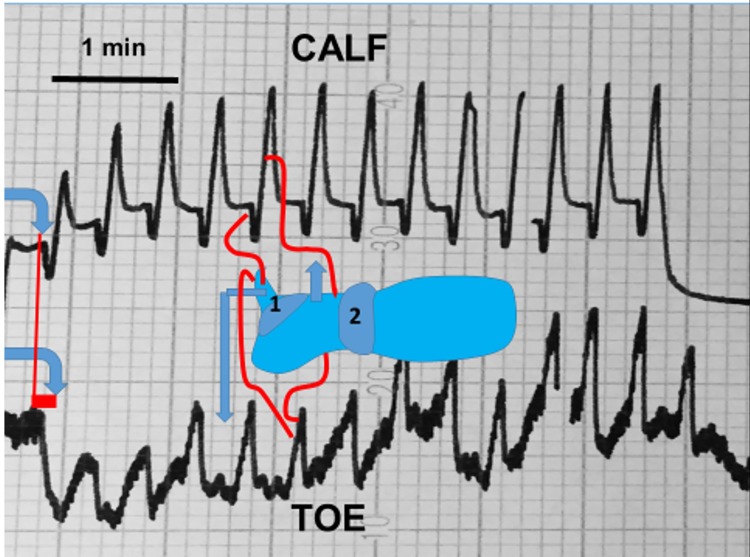
The circumference of the mid-toe and mid-calf was recorded during the arterial assist pump by strain gauge plethysmography in a patient with multifocal calf arteries changes. Number 1 and 2 denote sequentially inflated chambers in front-foot and calf. Blue lines depict strain gauges position. Red lines show time point when inflations began and toe and calf circumferences changed. Elongation of the gauge was read off on the recorder graph scale in mm. Increase in circumference was brought about by the in-flowing blood. Inflation of chamber 1 brought about a 2 sec decrease in calf and increase in toe circumference. Inflation of chamber 2 caused increase of both calf and toe circumference. Deflation was followed by return to the pre-support values. Note that each chamber inflation was followed by increase in toe or calf circumference. This might have been the effect of filling up with blood in occluded toe and calf veins.

The continuing recording of the toe and calf circumference during inflation of the arterial assist pump showed in all cases an increase, as long as the pump chamber was inflated (Fig 6). This was the effect of temporary accumulation of blood in the most peripheral vessels. At deflation of the arterial pump the circumference returned to the pre-compression values.

### The great saphenous vein blood pressure during arterial support

**Fig 7:**
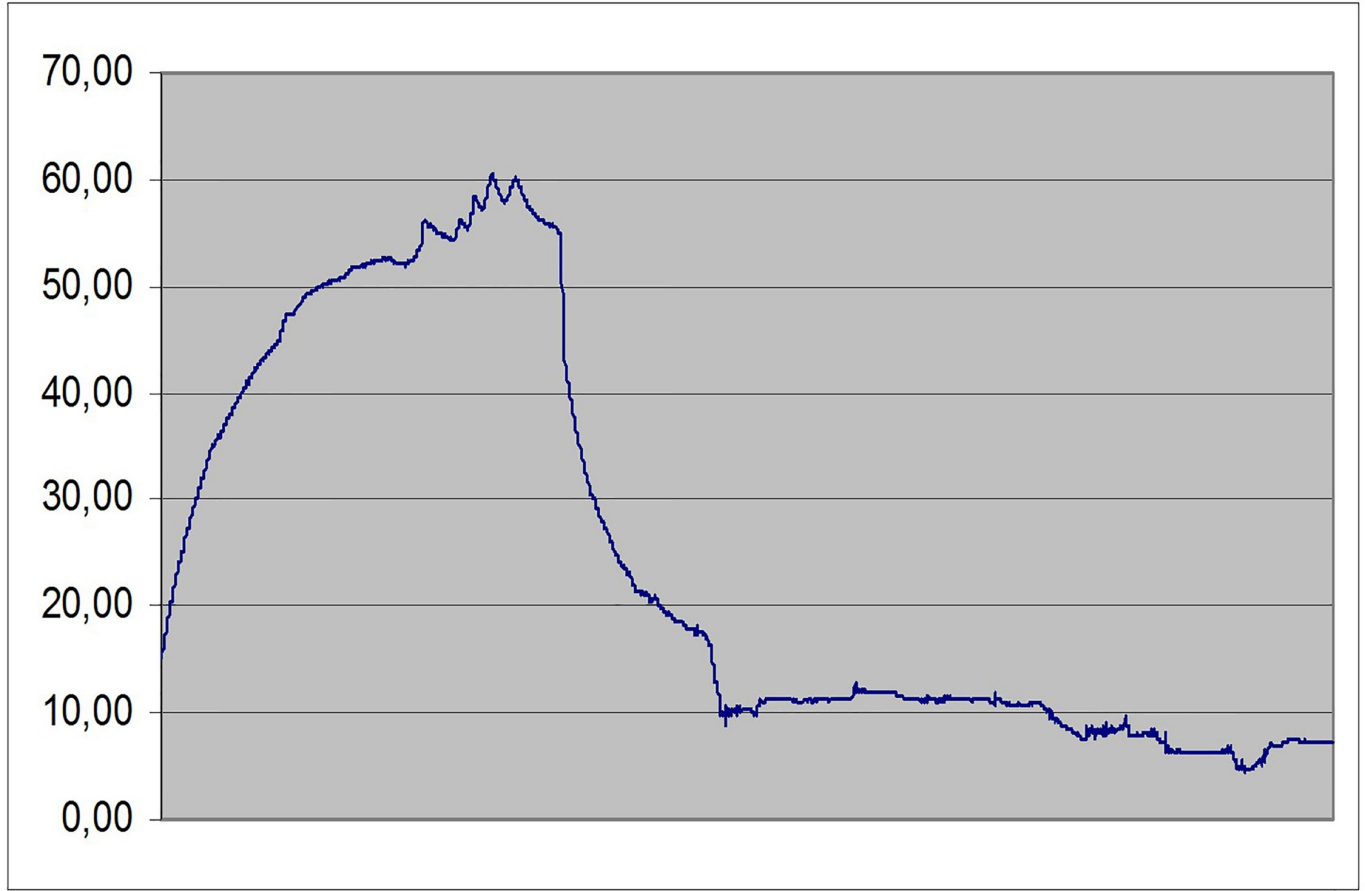
Blood pressure in the great saphenous vein (GSV) at the ankle level during arterial support pump inflations. Patient in a supine position. Increase of pressure during 10 inflations and fast decrease upon cessation of support. The pressure increase in the GSV proved stasis of venous blood. It may suggest also increase in venous capillaries.

In the 3 investigated subjects there was increase in pressure (Fig 7). This could be due to stasis of blood in the venous system caused by arterial pump venous obstruction. It returned to the initial values at pump cessation.

### Plethysmographic measurement of arterial blood inflow before and after arterial assist

**Fig 8:**
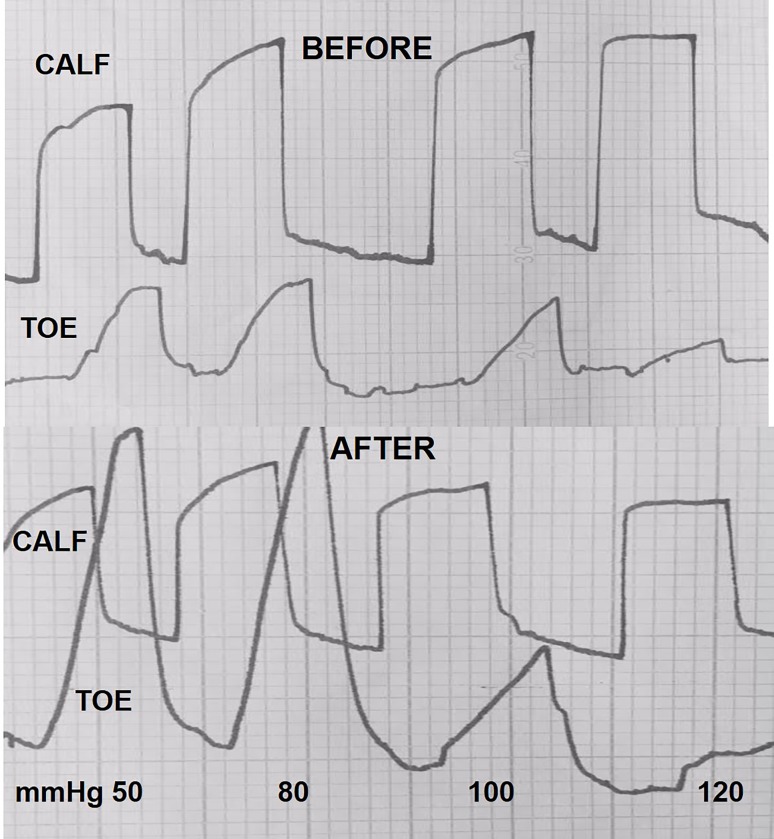
Venous occlusion plethysmography measuring arterial inflow at increasing occlusion pressures in a patient with multifocal changes in the calf arteries. Calf occlusion chamber was inflated to 50 to 120 mmHg pressures before and after 20 compressions of the designed arterial assist pump. Simultaneous recordings in the calf and big toe. Curves show increase of circumferences at each applied 0 mmHg occlusion pressures. Before compression: calf–fast rise of curves reaching same level at occlusion pressures up to 120mmHg, big toe- decreasing peak of circumference at occlusion pressures 100 and 120 mmHg. After compression: calf- no evident difference with before arterial assist, big toe- peaks higher than before assist. The difference may be due to the increase in capillary flow.

One-hour arterial assist evidently increased the toe blood inflow but not so much in the calf (Fig 8). The calculated mean one-minute inflow volume in the calf before IPC at plethysmographic obstruction pressure of 50mmHg was 25ml and remained at that level at 120mmHg. In the toe it was 2.5ml and 0.5ml, respectively. After IPC the calf inflow did not change significantly, whereas, there was increase in the toe to a mean 6.0ml at 50mmHg obstruction and 1.0ml at 120mmHg.

### The blood flow measurement before and after arterial assist for 2 years

**Fig 9:**
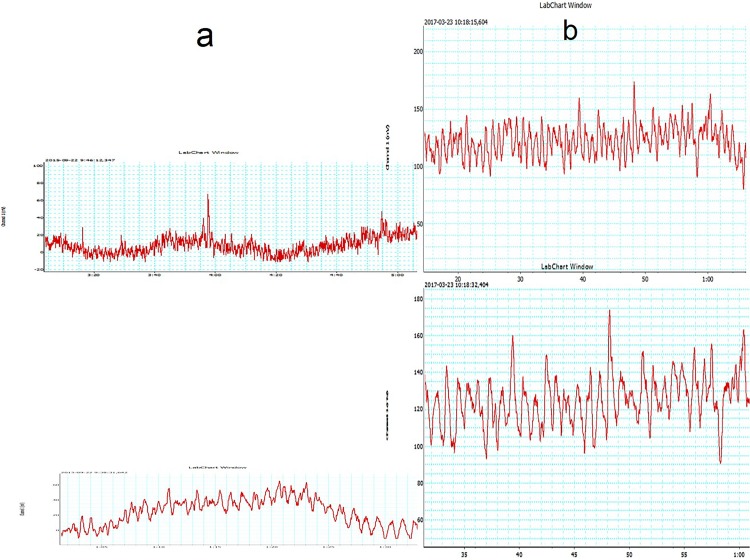
Laser Doppler recording of the big toe capillary flow velocity (in millivolts) in two patients with multifocal calf arteries occlusions after 2 years of the applied arterial assist. a-before and b- after long-term support. pre- and post-IPC scales adjusted. Evidently higher amplitudes after long-term therapy.

**Fig 10:**
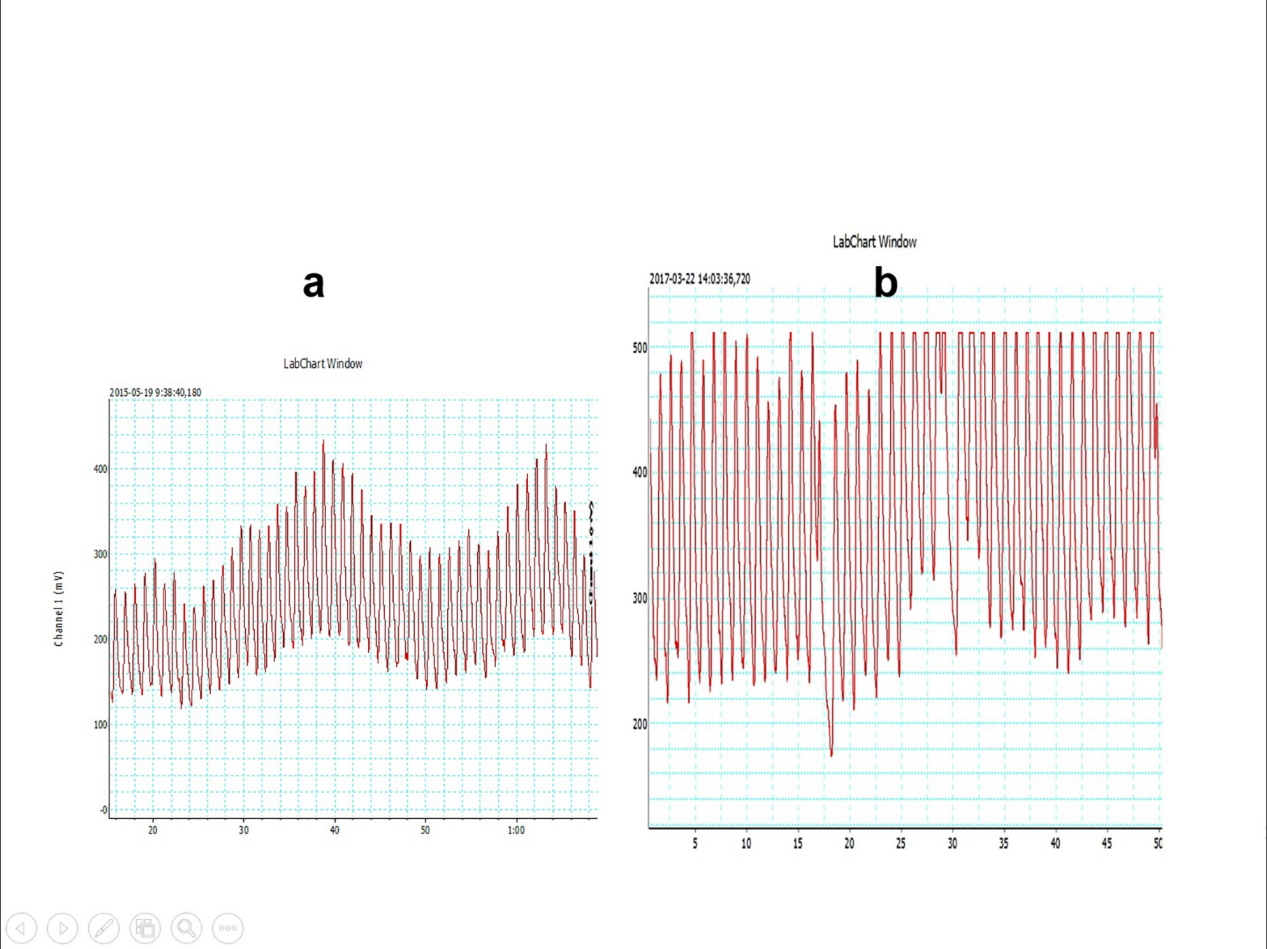
Laser Doppler recording of the big toe capillary flow velocity (in millivolts) in a patient with occlusion of the superficial femoral artery not suitable for reconstruction after 2 years of the applied arterial assist. a-before and b- after long-term assist. Pre- and post-IPC recording scales adjusted. Although the amplitudes before therapy were high there was an evident increase following the long-term assist.

Laser Doppler recordings of the big toe capillary flow velocity after 2 years arterial assist in 13 patients increased from 35±12 (5–65) to 125±57 (45–160) millivolts, p<0.05. Recordings of three patients are shown on Figs 9 and 10. There was evident increase in the capillary blood velocity in very advanced calf arterial changes as shown on Fig 9 but also in a case with high pre-therapy capillary flow shown on Fig 10.

### Plethysmographic measurement of arterial blood inflow after 2 years arterial assist

**Fig 11:**
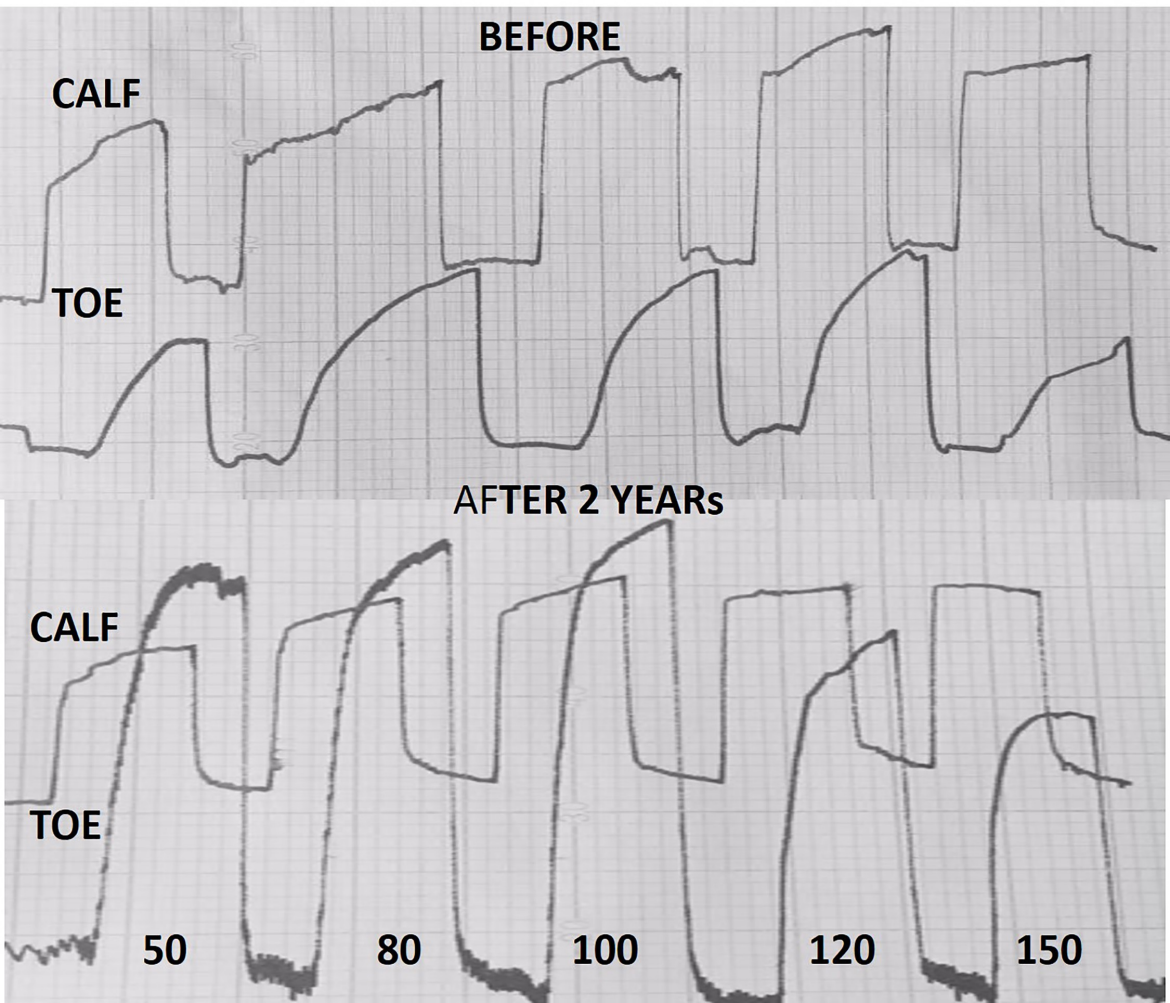
Calf and big toe plethysmography before and after 2 years of daily use of our arterial assist pump in a patient with calf multifocal arterial occlusions. The peak levels of calf arterial inflow do not show major difference before and after therapy. The peak levels of big toe inflow evidently higher than before therapy. Note no stop to arterial capillary flow at venous occlusion pressure of 150 mmHg.

Evidently increased big-toe capillary blood arterial inflow was observed in all cases (Fig 11). The calculated mean one-minute inflow volume in the calf before IPC (day 1) at plethysmographic obstruction pressure of 50mmHg was 25ml and remained at that level at 120mmHg. In the toe it was 2.5ml and 1.5ml, respectively. After IPC (two years) the calf inflow did not change significantly compared to the initial values, whereas, there was increase in the toe to a mean 6.0ml at 50mmHg obstruction and 4.0ml at 120mmHg (Table 1). Interestingly, even at 150mmHg obstruction pressure the inflow remained above 3ml.

**Table 1:** Plethysmography of calf and big-toe volume changes before and after 2 years of daily arterial pneumatic support (in ml).

Venous occlusion pressure (mmHg)	Volume Change	
	Before	After
Calf		
50	25+-5, (15–30)	25+-8, (15–36)
120	23+-5, (12–30)	25+-12 (15–46)
150	0	0
Big-toe		
50	2.5+-1.0 (0.5–3)	6.0+-2. 1(3–7)
120	1.5+-0.5(1–3)	4.0+-1.2 (5–0.5)
150	0	>3.0

### Clinical evaluation

Intermittent claudication distance reported by all patients increased by 20–120%. In some cases the distance of <100m increased to over 1000m. These data were obtained after a 2 years daily arterial support. The mean pretreatment ABI was 0.64 (range 0.4 to 0.76) and 0.7 (range 0.4 to 0.8) after one-hour assist (NS). The mean pretreatment TBI was 0.2 (range 0.05 to 0.2) and increased to 0.45 (range 0.1 to 0.55) after one-hour assist (p<0.05). The TBI after two years arterial assist was in the available 6 patients 0.6 (range 0.3 to 0.8) (p<0.05 vs pre-therapy).

## Discussion

Our hypothesis concerning the mechanism of pneumatic arterial support is that obstruction of the limb venous outflow by multiple pump-evoked venous occlusions would in a long period therapy expand the soft tissues network of perfusion vessels, and subsequently bring about persistent reactive hyperemia, restore supply of nutrients and evacuation of waste products. We observed at each of our pump inflation an increase in the toe arterial pressure, volume, capillary blood flow velocity and one-minute arterial inflow test. Long-term therapy showed persistence of the increased toe capillary flow velocity and arterial inflow. The TBI increased by approximately 20%. In contrast to the toe, the arterial inflow to the calf skin and muscles did not reveal improvement, however, there was evident prolongation of the claudication distance. The ABI did not increase, however, it may be presumed that dilatation of exchange vessels occurred also in the muscles, although it was not investigated in the present study [12,24].

Our findings concerning the soft tissue hyperemia after pneumatic assist remain in agreement with those of Delvis and Van Bemmelen, however, the interpretation of its mechanism is totally different [9,25]. They presumed that emptying of large veins provides space for arterial inflow, whereas we showed evidence of venous blood stasis resulting in persistent retrograde microvasculature dilatation bringing about decreased hydromechanic resistance to the arterial inflow. The difference between our and others’ result is that we used a slow inflation pump not evoking the venous-arteriolar reaction stopping the arterial inflow, whereas, they applied the ArtAssist pump striking soft tissues by rapidly inflated chambers for 0.6 sec. to 60-140mmHg bringing about temporary decrease in the arterial flow immediately after this fast inflation due to venous-arteriolar reaction. Decreased arterial inflow brought less blood in the veins.

Our arterial assist pump foot and calf chambers were slowly inflated for 5–6 sec to 120mmHg. Inflating first the foot and then the calf chamber increased the toe pulse amplitude in the phase of venous outflow obstruction followed by a brief total pulse stop when arteries became occluded. Then, there was a fast restoration of the pulse wave. The increase of pulse amplitude may be accounted for by obstruction of the venous outflow with the still remaining arterial inflow. In concert with the pulse amplitude rise the capillary flow velocity increased for seconds. During the continuing chamber inflations the capillary flow velocity was evidently increasing. Interestingly, it remained high for some minutes after assist cessation. It may be presumed that inflations repeated thousands of times resulted in suppressing the arteriolar vasoconstrictive activity that led to a persistent toe capillary dilatation. The importance of sufficient foot tissue capillary flow was shown by de Graaff who found that in arterial insufficiency of the leg the capillary pressure remains unscathed apparently through arteriolar vasodilatation compensating for the low arterial pressure [26]. According to Burton [27] it is a biophysical mechanism, in which no vascular neuro-vascular spasm may take place. His hypothesis was that the phenomenon depends just on the level of venous pressure.

The consequence of the venous blood pressure hit on the capillary endothelial cells could evoke gene expression effecting the vascular remodeling. The shear stress would be the main mechanical mediator [19]. The response of the endothelial cells resulting in opening more capillaries and their dilatation would be the physiological response to the continuing mechanical stress.

In conclusion, the arterial assist pump we applied in patients with leg ischemia brought about increase in the toe capillary flow, generated long lasting dilatation of big-toe capillaries and clinically extension of painless walking distance. The physiological effect of arterial assist is dilatation of capillaries by the retrograde force generated by obstruction of venous outflow. Doppler blood velocity test and venous occlusion plethysmography (arterial inflow) of the big toe and calf provided evidence for these hemodynamic effects. The long-term usage of the assist pumps results in persistent dilatation of the microvasculature. The crucial factor of rhythmic multiple venous outflow obstructions causing retrograde capillary dilatation should be taken into account in designing the new effective assist devices.
